# SIRT1/3/6 Landscape of Human Longevity: A Sex- and Health-Stratified Pilot Study

**DOI:** 10.3390/biology14101353

**Published:** 2025-10-02

**Authors:** Ulduz Hashimova, Igor Kvetnoy, Aliya Gaisina, Khatira Safikhanova, Ekaterina Mironova, Irana Galandarli, Lala Hasanli

**Affiliations:** 1 Institute of Physiology Named After Academician Abdulla Garayev, Ministry of Science and Education of the Republic of Azerbaijan, AZ1100 Baku, Azerbaijan; aliyagaisina@hotmail.com (A.G.); khatira.safikhanova.74@mail.ru (K.S.); irana900@mail.ru (I.G.); 2 Center of Molecular Biomedicine, Saint-Petersburg Research Institute of Phthisiopulmonology, 191036 Saint Petersburg, Russia; igor.kvetnoy@yandex.ru (I.K.); katrine1994@mail.ru (E.M.); 3 Medical Institute of Saint-Petersburg State University, 199034 Saint Petersburg, Russia; 4 Medical Faculty, Nakhchivan State University, AZ7000 Nakhchivan, Azerbaijan; lalealimli@gmail.com

**Keywords:** sirtuins, protein-to-mRNA ratio, healthy ageing, sex, cardiovascular status

## Abstract

Sirtuins (SIRT1–SIRT7) are NAD^+^-dependent deacetylases that link cellular energy status to chromatin maintenance, mitochondrial function and inflammatory signaling. While modulation of SIRT1, SIRT3 and SIRT6 extends lifespan in model organisms, evidence in extreme-age humans is scarce. We quantified protein and mRNA levels, and protein-to-mRNA ratios for SIRT1, SIRT3 and SIRT6 in buccal epithelial cells obtained from healthy young adults, middle/late-aged individuals and nonagenarians/centenarians residing in a longevity-enriched region of south-eastern Azerbaijan. The cohort comprised 23 participants, stratified by sex and cardiovascular disease (CVD) status (5 per sex/CVD subgroup). This design allows us to: (1) define a baseline “sirtuin profile” of healthy longevity, (2) evaluate the impact of CVD as a prevalent age-related pathology, and (3) explore potential sex-specific modulation. These findings establish an initial human framework linking sirtuin translational control to healthy ageing and cardiovascular health.

## Simple Summary

Rapid demographic ageing is transforming economies and health systems, compelling WHO’s Healthy Ageing framework to prioritize the preservation of older adults’ functional autonomy and social engagement. Achieving this goal hinges on a deep understanding of the biological mechanisms of ageing—which reflect complex interactions between genetic predisposition, environment and lifestyle—since genetic factors play a modest role in reaching age 70 but become increasingly influential in extreme longevity. Identifying reliable molecular biomarkers is therefore critical for predicting health outcomes, guiding preventive strategies and developing targeted therapies. In this pilot study, we assayed the activity of sirtuin enzymes—master regulators of key aging processes: energy metabolism, genome stability and inflammatory homeostasis—to establish simple, predictive biomarkers and actionable intervention targets for sustaining functional autonomy across the lifespan.

## 1. Introduction

Population aging is one of the most significant social phenomena of the 21st century, fundamentally reshaping all determinants of the post-industrial societies. According to current projections, the global population of older adults will double by 2050, reaching 1.5 billion, with the proportion of individuals aged 65 and over rising from 6% in 1990 to 16% by mid-century [1].

As natural generational transition is underway, it is giving rise to a new cohort of longevity “champions,” shaped not by pre-industrial rural lifestyles, but post-war reconstruction, strong social cohesion, and a lifelong pursuit of education and self-betterment. Unlike predecessors, these individuals—predominantly born in the late 1920s and 1930s, as well as the following baby boomer generation of the 1950s—are not merely passive survivors of historical challenges, but active participants in a transformative era, redefining aging as an active, purposeful phase of life [2,3,4].

Against this evolving demographic and cultural background, scientific interest increasingly focuses on the molecular and physiological mechanisms for not only extended lifespan, but healthy aging [3,4,5,6].

Azerbaijan has traditionally been regarded as one of the world’s longevity “hotspots”—particularly in its rural and mountainous regions, where ancestral lifeways, strong family bonds, and favorable environmental conditions have long prevailed [7]. Comprehensive cross-cohort studies have revealed familial clustering of longevity in this population and identified a stable phenotype characterized by preserved cardiovascular health, absence of age-associated dementia, and sustained sex hormone levels well into advanced age [7,8]. Notably, despite the generational shifts from traditional to more industrial and urbanized lifestyles, the proportion of residents aged ≥ 90 years in Azerbaijan increased by 21%, indicating that genetic, socio-cultural and environmental factors continue to sustain exceptional longevity [9].

In this context, identifying molecular markers and regulatory pathways that reflect and potentially mediate these population-level aging dynamics has become a key scientific priority [10,11,12].

Aging has been conceptualized through multiple theoretical frameworks: mutation accumulation; the oxidative/mitochondrial free radical theory; antagonistic pleiotropy; disposable soma; proteostasis collapse; hyperfunction/programmatic aging; misrepair accumulation theory; and the information theory of aging, which posits a progressive loss of epigenetic and transcriptional fidelity—to name only a few, with more models likely to emerge [13,14,15,16,17,18,19,20,21,22]. Regardless of the starting premise—damage, trade-offs, quasi-programs, or information decay—they converge on a shared set of molecular events collectively known as the Hallmarks of Aging: genomic instability, telomere attrition, epigenetic alterations, loss of proteostasis, mitochondrial dysfunction, deregulated nutrient sensing, cellular senescence, stem-cell exhaustion, altered intercellular communication, etc. [23]. Within the geroscience paradigm, sirtuins—NAD^+^-dependent class III histone/protein deacetylases, with some isoforms exhibiting desuccinylase, demalonylase, and deglutarylase activities—occupy a central position at the nexus of longevity core domains: informational integrity (via SIRT1/6), mitochondrial bioenergetics and redox homeostasis (via SIRT3), and coordinated nutrient–stress sensing (the IIS–mTOR–AMPK–NAD^+^ axis) [24,25,26,27,28]. Rather than serving as passive markers of aging, sirtuins function as proactive regulators of resilience and lifespan. Across mammalian models, manipulating sirtuin activity genetically or via small-molecule activators improves metabolic, cardiac, and neural stress resistance and modulates longevity in a context- and sex-dependent manner, highlighting SIRT1, SIRT3, and SIRT6 as tractable anti-aging targets [29,30,31]. To date, however, evidence from human cohorts—especially nonagenarians and centenarians—remains limited, but also robust. Human genetic evidence supports the mechanistic role of sirtuins in healthy aging: carriers of certain SIRT6 minor alleles show ≥5-year survival advantage [32], while rare centenarian-specific SIRT6 variants enhance DNA repair and suppress retrotransposon activity [33]. SIRT1 protein levels correlate positively with age and may compensate for oxidative stress in elderly populations [34]. Together, these results highlight the biological plausibility and amenability of sirtuins as therapeutic targets for promoting longevity and enhancing systemic resilience [35,36].

Given that context, the present pilot study aimed to profile SIRT1, SIRT3, and SIRT6 expression in an Azerbaijani longevity cohort, examining sex and cardiovascular-health differences and evaluating mRNA–protein ratios as indicators of post-transcriptional regulation linked to functional resilience.

## 2. Materials and Methods

The study was performed on buccal epithelium (BE), which was selected as a highly informative, non-invasive surrogate tissue for evaluating gene and protein expression in aging research [37,38,39].

### 2.1. Study Population

This cross-sectional survey was conducted in the Lankaran region of Azerbaijan. The study enrolled ten very old adults (VO, ≥90 years; mean ± SD: 95.7 ± 3.7 years; 5 men, 5 women), along with thirteen of their first-degree relatives serving as comparative groups: a middle-to-late adulthood group (MA, 51–84 years; mean ± SD: 62.7 ± 9.8 years; 5 men, 5 women) and a young adulthood group (YA, 18–29 years; mean ± SD: 24.0 ± 5.1 years; 1 man, 2 women). The age of the VO participants was verified using national identity documents.

All individuals were examined and sampled in their usual place of residence. Clinical inspection immediately prior to sampling ensured intact, inflammation-free buccal mucosa in each participant, fulfilling the primary inclusion criterion for biological sampling. None of the participants exhibited clinical signs of dementia or diabetes, nor reported any related diagnoses or medication use.

For study purposes, each participant was classified as either CVD − (no cardiovascular disease) or CVD + (documented ischemic heart disease and/or stage I–II arterial hypertension), based on medical history and current treatment.

Written informed consent was obtained from all participants. The study protocol was approved by the Ethics Committee of the Institute of Physiology named after Academician Abdulla Garaev.

### 2.2. Sample Collection

Buccal epithelial cells were obtained by gently brushing the inner cheek mucosa with a sterile cytology brush. The collected cells were immediately rinsed in phosphate-buffered saline (Thermo Fisher Scientific, Waltham, MA, USA) to remove debris and then placed in 4% paraformaldehyde (Sigma-Aldrich, St. Louis, MO, USA) for transport at 4 °C and processed (completion of fixation or RNA extraction) within 8–10 h of collection.

Prior to enrollment, all participants underwent a dental examination to confirm the absence of any dental or buccal pathology.

### 2.3. Immunofluorescent Staining

Fixed buccal-epithelial cells were permeabilized in 0.1% Triton X-100 (Thermo Fisher Scientific, Waltham, MA, USA) for 10 min and blocked in 5% bovine serum albumin (Thermo Fisher Scientific, Waltham, MA, USA) for 30 min. Samples were incubated overnight at 4 °C with primary antibodies against SIRT1 (1:100; Cambridge, MA, USA), SIRT3 (1:50; Cambridge, MA, USA), and SIRT6 (1:100; Cambridge, MA, USA). After three PBS washes, slides were incubated for 1 h at room temperature with Alexa Fluor-conjugated secondary antibodies (Alexa 488, Alexa 555, or Alexa 567; 1:1000; Cambridge, MA, USA) and then counterstained with Hoechst 33258 (1 µg/mL; St. Louis, MO, USA). Images were acquired on an inverted Olympus Fluoview FV300/IX70 confocal microscope (Olympus Corporation, Tokyo, Japan) (63× oil-immersion objective) under identical laser power, gain, and offset settings.

### 2.4. Image Quantification

Mean fluorescence intensity was measured in five randomly selected fields per sample; each field was analyzed in triplicate and the three measurements averaged to yield a single intensity value per sample.

### 2.5. Morphometric Analysis

Ten random fields per sample were captured and processed with Videotest-Morphology 5.2. Immunopositive area (%) was calculated as:

Percentage of immunopositive staining = (Area of immunopositive staining ÷ Total cellular area) × 100%

### 2.6. RNA Extraction and Quantitative Real-Time PCR

Total RNA was subsequently isolated from buccal epithelial cell pellets using the RNeasy Mini Kit (Qiagen, Valencia, CA, USA) with on-column DNase digestion. First-strand cDNA was synthesized from 1 µg of RNA using the RevertAid First Strand Kit (Thermo Fisher Scientific, Waltham, MA, USA). Quantitative PCR was performed in triplicate on a QuantStudio 5 system (Thermo Fisher Scientific, Waltham, MA, USA) using TaqMan™ Gene Expression Assays (Applied Biosystems, Foster City, CA, USA) for SIRT1, SIRT3, SIRT6, and GAPDH, and in parallel on a CFX96 Real-Time PCR Detection System (Bio-Rad Laboratories, Hercules, CA, USA) with the QuantiFast SYBR Green PCR Kit (Qiagen, Valencia, CA, USA) employing primers spanning exon–exon junctions. Relative gene expression was calculated by the 2^–ΔΔCt^ method, using GAPDH as the endogenous control.

### 2.7. Statistical Analysis

Statistical analyses were conducted in IBM SPSS Statistics v29.0.2.0. Given the pilot nature of the study (*n* = 23; 5–10 participants per subgroup), nonparametric methods were applied throughout with a two-sided significance threshold of *α = 0.05*. Descriptive statistics for protein levels, mRNA expression, and protein-to-RNA ratios (PTRs) across the three age cohorts are presented as medians and interquartile ranges (Q1–Q3). Relative changes (%) calculated as *Δ*% = (*value*_2_ − *value*_1_)/*value*_1_ × 100. Pairwise comparisons between two groups were performed using exact Mann–Whitney U tests, with effect sizes calculated as *r =* |Z|/√(*N*_1_ + *N*_2_); *r* ≥ 0.50 denotes a large effect. Overall differences among three or more groups were evaluated by Kruskal–Wallis tests, followed by Dunn’s post hoc procedure for multiple comparisons. Relationships between continuous variables were assessed via Spearman’s rank-order correlation (*ρ*), with 95% confidence intervals derived from 5000 bootstrap samples; partial correlations controlling for age, cardiovascular disease status, or sex were also computed.

## 3. Results

### 3.1. Age-Dependent Dynamics of Expression and Translational Efficiency of SIRT1, SIRT3, and SIRT6

Studies have shown a clear age-dependent decline in protein expression and mRNA levels for SIRT6, SIRT3, and SIRT1 (Table 1 and Table 2). Protein abundances decrease monotonically from young adulthood (YA) through middle/late adulthood (MA) to very old age (VO) in the hierarchy SIRT6 > SIRT3 > SIRT1, with overall YA→VO declines of –63.8%, –57.2%, and –56.1%, respectively. mRNA dynamics follow the same sequence (YA→VO: –69.6%, –54.8%, –42.1%), with the steepest transcriptional drop in SIRT6 (–51.7%) and the smallest in SIRT1 (–24.0%) during YA→MA; in MA→VO, SIRT6 mRNA continues to fall more sharply (–36.4%) than SIRT3 (–20.0%) and SIRT1 (–24.7%). PTR ratios reveal unexpected age-related translation efficiency patterns: for SIRT3, PTR shows only a modest decline across age groups (1.85→1.79→1.70), indicating proportional regulation of protein and transcript; SIRT1 PTR decreases more sharply in middle age (1.98→1.36→1.33), indicating progressively reduced translational efficiency; for SIRT6, PTR first rises to a peak at MA (1.81→2.57) despite mRNA reduction, then falls to 2.08 in VO, indicating a robust mid-life translational compensatory response that partially wanes in the oldest cohort. Thus, our data demonstrate that PTR preservation in the VO versus YA group follows the hierarchy SIRT6 (118.2%) > SIRT3 (91.9%) > SIRT1 (67.2%), underscoring that SIRT6 translation efficiency is most resilient to aging.

### 3.2. Sex-Dependent Dynamics of Expression and Translational Efficiency of SIRT1, SIRT3, and SIRT6

Age stratification revealed a sex-related divergence in SIRT activity trajectories (Table 3 and Table 4). Both sexes exhibited age-dependent declines, but with markedly different kinetics and amplitudes. In men, S-SIRT3 and S-SIRT6 levels declined gradually from the young-adult (YA) to middle/late-aged (MA) stages, while S-SIRT1 exhibited a more pronounced drop. From MA to very-old (VO), all six markers—S-SIRT1, S-SIRT3, S-SIRT6 and their corresponding mRNA levels—fell by several fold (Kruskal–Wallis *p* < 0.01, ε^2^ more than 0.60, indicating a large effect size). Post-hoc Dunn’s tests with Bonferroni correction confirmed that most markers showed their largest median difference between the YA and VO groups (adjusted *p* = 0.017). Protein-to-mRNA ratios for SIRT1 mirrored this downward trajectory from YA through MA to VO, whereas PTR for SIRT3 and SIRT6 rose in MA and only slightly declined at VO (Table 4).

In contrast, women showed a pronounced decline from young adulthood to middle age —likely driven by hormonal shifts—followed by marker stabilization: median S-SIRT3 and S-SIRT6 in very old remained >2.0 (only 20–30% below YA), and mRNA-SIRT6 at 95–102 years matched MA levels. No marker achieved statistical significance across age groups (*p* > 0.05), and effect sizes were modest (ε^2^ = 0.12–0.18), reflecting a smoother, more gradual trajectory. Women also exhibited divergent post-transcriptional regulation: PTR-SIRT1 follows a biphasic trajectory with attenuation in midlife and recovery in the very old, whereas PTR-SIRT3 and PTR-SIRT6 rises steadily with age and remains elevated into extreme old age.

Across all pairwise compare, women consistently exhibited higher marker levels than men, with the greatest differences in the very-old group: median values were roughly 1.3- to 1.5-fold higher, and every Mann–Whitney comparison produced a large, standardized effect calculated as *r* = *Z*/√(*n*_1_ + *n*_2_) (0.53 ≤ *r* ≤ 0.68). Nevertheless, the small sample size (*n*_1_ = *n*_2_ = 5) kept the corresponding *p*-values just above the conventional α = 0.05 threshold (0.09 ≤ *p* ≤ 0.10), indicating limited statistical power rather than a lack of biological divergence.

### 3.3. Health Status Correlation

All data are presented in Table 5 and Table 6.

Comparison of the CVD– and CVD+ groups showed that all three sirtuin proteins were markedly lower in CVD+, with the steepest depletion in S-SIRT1 (−78%), followed by S-SIRT6 (−73%) and S-SIRT3 (−71%). The Mann–Whitney U test yielded *p* = 6.3 × 10^−5^ and a rank-biserial effect size *r* = 0.84, i.e., a large effect by conventional criteria.

On the transcript level the order was reversed (SIRT6 > SIRT1 > SIRT3): mRNA-SIRT6 showed the largest deficit (−66%), followed by mRNA-SIRT1 (−42%) and mRNA-SIRT3 (−27%). Nevertheless, all three mRNAs remained strongly associated with CVD (0.001 ≤ *p* ≤ 0.003; *r* ≈ 0.63–0.69). Translational efficiency tracked these shifts: PTR-SIRT1 and PTR-SIRT3 collapsed by roughly half (−55% and −59%; *p* = 6.3 × 10^−5^ and 2.2 × 10^−4^; *r* = 0.84 and 0.77), mirroring their protein losses, whereas PTR-SIRT6 declined only slightly and non-significantly (−9%, *p* = 0.56, *r* = 0.11).

Spearman correlation analysis confirmed the pattern (ρ = −0.86 for S-SIRT1/3 and ρ = −0.67 for S-SIRT6, *p* < 0.001). After adjustment for age and sex, the correlations stayed virtually unchanged (partial ρ ≈ −0.98, −0.90 and −0.74; *p* < 0.001), underscoring their independence from sex and only a moderate linkage to chronological age.

Stratifying the cohort by age and cardiovascular status further revealed “healthy-ageing” profile: CVD-negative very-old adults preserve roughly 70% of youthful SIRT1/3, about 60% of SIRT6, and display 25–30% higher SIRT3 and SIRT6 protein-to-mRNA ratios—an internal benchmark of molecular resilience that contrasts sharply with the deficits observed in their CVD-positive peers (Table 6).

## 4. Discussion

Longevity is a multi-factorial phenomenon shaped by the complex interplay between genotype and environment, with genetic influence seemingly becoming more decisive at advanced ages [40,41]. Rapid progress in preventive medicine, ever-more sophisticated population-risk management and unprecedented improvements in hygiene, living conditions and workplace standards have undoubtedly pushed life expectancy upward. Yet a paradox persists: modern technology does not eliminate stressors; it merely reconfigures them, so that reaching extreme ages is still the exception rather than the rule and is achieved while carrying an ever-growing, cross-generational load of external and internal challenges [3]. This reality highlights the strategic need to identify the core biological mechanisms that can offset cumulative organismal wear and thereby preserve functional autonomy at the limits of the human lifespan.

Against this backdrop, the NAD^+^-dependent sirtuin family—most prominently SIRT1, SIRT3 and SIRT6—emerge as an evolutionarily conserved yet readily actionable control node of cellular health [25,26,27,28,42,43]

Their strong association with lifespan extension in multiple species, coupled with their near-ubiquitous expression and broad spectrum of regulatory functions, makes them especially compelling targets for interventions aimed at prolonging healthy human life [24]. In brief, SIRT1 acts as a nuclear–cytoplasmic energy sensor that coordinates vascular tone and restrains pro-inflammatory signaling [44]; SIRT3, the predominant mitochondrial deacetylase, maintains organelle integrity by fine-tuning oxidative phosphorylation, detoxifying reactive oxygen species and promoting mitophagic turnover [45]; and SIRT6 serves as a chromatin gatekeeper, orchestrating base-excision DNA repair, telomere maintenance and suppression of NF-κB-driven transcriptional programs [46]. Together, these sirtuins form a genomic defense triad—preserving DNA and epigenomic integrity, minimizing misrepair, and supporting the fidelity of cellular identity, thus buffering the molecular tipping points beyond which aging and age-related diseases emerge.

Aging studies in both humans and animals reveal a progressive global loss of transcriptional control and uncoupling of transcript and protein levels, reflecting a decline in information integrity—the fidelity of genetic information transfer from transcript to proteome [47,48,49,50]. This pattern is strongly context-dependent, varying by gene identity, sex, and tissue type [51,52,53]. To compensate, cells engage translational buffering mechanisms, selectively upregulating the translation of specific mRNAs to offset transcript-level declines and preserve essential proteome functions—a strategy that helps maintain cellular homeostasis in ageing. Notably, translational buffering is not a universal process but is selectively engaged for a subset of genes whose protein products are critical for cellular survival and function [54,55]. Whether sirtuins are prioritized in this compensatory hierarchy remains to be determined.

Profiling all three sirtuins in parallel enabled our pilot study to define a composite “sirtuin phenotype” of ageing, that provides insight into how the nuclear (SIRT1), mitochondrial (SIRT3) and chromatin (SIRT6) branches of the pathway co-adjust across sex and baseline health status in ways that favour longevity.

Our study has shown that although SIRT1, SIRT3 and SIRT6 levels predictably fell with age, the magnitude of these declines was significantly influenced by both sex and baseline cardiovascular health.

Women retained higher absolute pools of S-SIRT1 and S-SIRT3 and exhibited a smaller loss of S-SIRT6 than men; their protein-to-mRNA ratios—our proxy for translational efficiency—rose by ≈30% for SIRT3 and SIRT6, whereas the male increase was modest. This pattern is consistent with hormone-dependent regulation: estrogens acting through ER-α/β up-regulate SIRT1 transcription in endothelial and cardiac cells [56], via E_2_-ERα boost SIRT3 expression and mitochondrial targeting, enhancing oxidative phosphorylation, antioxidant defenses, and mitophagy for improved mitochondrial health [57,58] and enhance SIRT6 activity by shielding critical acetyl-lysine residues, whereas androgens are neutral or even suppressive [59,60]. Moreover, a pre-clinical study demonstrated that female mice retain markedly higher renal and circulating SIRT6 after ischaemia–reperfusion injury than males; ovariectomy abolished this advantage, whereas 17β-oestradiol supplementation restored it—direct evidence of an estrogen-dependent protective axis [61].

In addition to hormone-dependent regulation, sex-specific, hormone-independent mechanisms—mediated by X-chromosome dosage and epigenetic regulation—may influence sirtuin expression and function. Although direct evidence is lacking, key X-linked epigenetic regulators, such as KDM6A (UTX), and sex-biased DNA methylation and histone modifications shaped by X-linked gene activity could directly or indirectly modulate sirtuin pathways [62,63,64] Additionally, sirtuins interact with FOXO factors (e.g., SIRT3-FOXO3A), a key axis in stress resistance and survival [65,66]. Epidemiological studies further show that FOXO3’s protective effects are stronger in women, while SIRT1’s impact may be greater in men, highlighting complex sex-specific regulatory networks beyond hormones [67,68].

Taken together, these hormone- and non-hormone-driven divergences provide a mechanistic context for the sex-specific maintenance of sirtuin translational efficiency—and thus mitochondrial and chromatin resilience—observed in our female cohort and may further account for their higher life-expectancy and common predominance among the oldest-old [69].

Our findings likewise showed that the presence of cardiovascular disease (CVD) reshapes the sirtuin axis far more dramatically than chronological aging and sex. We observed a decline in SIRT1, SIRT3, and SIRT6 levels, broadly consistent with a ~50% reduction in SIRT1 reported in ischemic heart disease cohorts [70,71,72] and a ~35% decline in SIRT3 under pressure-overload conditions [73]. Partial Spearman correlation analysis—adjusted for age and sex—confirmed a direct association between lower sirtuin levels and CVD (ρ ≈ –0.98 for SIRT1, –0.90 for SIRT3, and –0.74 for SIRT6), echoing meta-analytic data that position low circulating SIRT1 among the strongest molecular correlates of adverse cardiac events [74]. Thus, SIRT1 emerges as the principal “culprit” node, with SIRT3 exhibiting an intermediate response.

In contrast, SIRT6 behaves differently: although its absolute protein level fell by ~73%, the protein-to-mRNA ratio remained virtually unchanged (–9%, *p* = 0.56). This pattern exemplifies translational buffering—specifically the “offsetting” mode—whereby cells upregulate translation of selected proteins to maintain critical functions despite drops in mRNA levels; in this case, ensuring essential chromatin maintenance when transcription falters [55]. Consequently, the so-called SIRT6 “paradox” is more accurately framed as an emergency protective buffer, rather than a pathological driver.

Finally, stratification of our cohort by CVD status revealed a probabilistic profile of successful ageing: in CVD-free participants, approximately 70% of youthful circulating SIRT1 and SIRT3—and about 60% of SIRT6—protein levels were preserved, echoing experimental and clinical evidence that maintained SIRT1/SIRT3 activity and enhanced SIRT6 translation mark healthy longevity. Moreover, instead of the protein–mRNA decoupling often described in ageing tissues, these individuals exhibited a ~30% increase in PTR for SIRT3 and ~25% for SIRT6. This pattern mirrors murine models in which enhanced SIRT6 translational efficiency prolongs median lifespan by ~15% [75]; and human genetic analyses linking SIRT3 variability to exceptional survival [12,76], positioning boosted SIRT3 and SIRT6 translation not merely as a compensatory response but as a fundamental mechanism of functional longevity. Overall, our data support the concept that preserved translational compensation, rather than absolute abundance alone, underpins healthy aging [55].

This pilot study is the first to profile SIRT1, SIRT3 and SIRT6 across sex, age and cardiovascular health, defining a unified “sirtuin phenotype” that integrates nuclear energy sensing, mitochondrial integrity and chromatin maintenance as axes of cellular resilience. Although based on a small, cross-sectional cohort, the large and internally consistent effect sizes pave the way for longitudinal studies to validate sirtuin translational efficiency as a predictive biomarker of healthy ageing and cardiovascular resilience across sexes and as a target for sirtuin-modulating interventions aimed at extending healthspan.

## 5. Conclusions

This pilot investigation demonstrates that preserved translational compensation of SIRT1, SIRT3 and SIRT6—evidenced by maintained protein-to-mRNA ratios despite declining absolute levels—may serve as a hallmark of functional longevity. By quantifying protein and mRNA across age groups, sexes and cardiovascular health status, we revealed robust effect sizes and distinct translational efficiency signatures for each subgroup. Although limited by its small, cross-sectional design, our study highlights sirtuin translational control as both a predictive biomarker of healthy ageing and cardiovascular resilience and a viable intervention target. These findings warrant validation in larger, longitudinal cohorts and mechanistic trials to assess whether boosting sirtuin translation can preserve autonomy and extend healthspan.

## 6. Limitations

As a pilot study, the modest sample size and incomplete age coverage limit statistical power and preclude a fully continuous age gradient. Consequently, some non-significant *p*-values may reflect underpowered subgroup analyses rather than the absence of biological effects. The cross-sectional design further constrains causal inference. Finally, although PTR provides an integrated measure of transcriptional and translational dynamics, the potential influence of post-translational turnover cannot be fully excluded.

## Figures and Tables

**Table 1:** Sirtuin 1/3/6 Profiles of Aging Cohorts.

Marker	YA	MA	VO
Protein levels (S-)			
S-SIRT1	2.23 [2.21; 2.31]	1.41 [0.80; 2.02]	0.98 [0.41; 1.58]
S-SIRT3	2.90 [2.88; 2.97]	1.79 [1.34; 2.11]	1.24 [0.54; 2.00]
S-SIRT6	3.70 [3.65; 3.75]	2.52 [2.00; 2.69]	1.34 [0.61; 2.11]
mRNA levels			
mRNA–SIRT1	1.21 [1.13; 1.26]	0.93 [0.88; 1.07]	0.70 [0.51; 0.83]
mRNA–SIRT3	1.59 [1.57; 1.60]	0.90 [0.78; 1.35]	0.72 [0.59; 0.81]
mRNA–SIRT6	2.07 [2.04; 2.09]	0.99 [0.92; 1.07]	0.63 [0.30; 0.96]
Protein-to-mRNA ratio (PTR)			
SIRT1/mRNA	1.98 [1.84; 2.09]	1.36 [0.91; 1.73]	1.33 [0.81; 1.90]
SIRT3/mRNA	1.85 [1.83; 1.88]	1.79 [1.06; 2.18]	1.70 [0.90; 2.47]
SIRT6/mRNA	1.76 [1.76; 1.80]	2.57 [2.36; 2.64]	2.08 [1.94; 2.22]

Values are presented as median [Q1; Q3] for each marker in three age groups: young adults (YA), middle/late- aged (MA) and very old (VO).

**Table 2:** Age-Related Percentage Change (reference = median of YA).

Marker	MA vs. YA (%)	VO vs. MA (%)	VO Remaining vs. YA (%)
Protein levels (S-)			
S-SIRT1	−36.8	−30.5	43.9%
S-SIRT3	−38.3	−30.8	42.8%
S-SIRT6	−31.9	−46.8	36.2%
mRNA levels			
mRNA–SIRT1	−23.2	−24.7	57.9%
mRNA–SIRT3	−43.4	−20.0	45.3%
mRNA–SIRT6	−51.2	−36.4	30.4%
Protein-to-mRNA ratio (PTR)			
SIRT1/mRNA	−31.3	−2.2	67.2%
SIRT3/mRNA	−3.2	−5.0	91.9%
SIRT6/mRNA	+46.0	−19.1	118.2%

Value calculated as MA vs. YA = (MA − YA)/YA ∗ 100, VO vs. MA = (VO − MA)/MA ∗ 100, VO remaining vs. YA = VO/YA ∗ 100.

**Table 3:** Sirtuin 1/3/6 Expression Profiles by Age Cohort and Sex.

Marker	YA (♂ + ♀)	MA ♂	MA ♀	VO ♂	VO ♀
Protein levels (S-)					
S-SIRT1	2.23 [2.21; 2.31]	0.81 [0.77; 2.02]	1.99 [0.83; 2.10]	0.41 [0.41; 0.45]	1.60 [1.52; 1.67]
S-SIRT3	2.90 [2.88; 2.97]	1.34 [1.34; 2.04]	2.00 [1.57; 2.13]	0.58 [0.50; 0.60]	2.01 [1.67; 2.10]
S-SIRT6	3.70 [3.65; 3.75]	2.55 [2.34; 2.69]	2.50 [1.89; 2.67]	0.65 [0.59; 0.67]	2.11 [2.10; 2.14]
mRNA levels					
mRNA–SIRT1	1.21 [1.13; 1.26]	0.97 [0.90; 1.10]	0.91 [0.87; 0.94]	0.51 [0.49; 0.59]	0.83 [0.80; 0.83]
mRNA–SIRT3	1.59 [1.57; 1.60]	1.22 [0.88; 1.40]	0.83 [0.75; 0.92]	0.60 [0.56; 0.65]	0.82 [0.79; 0.83]
mRNA–SIRT6	2.07 [2.04; 2.09]	0.99 [0.99; 1.00]	1.01 [0.80; 1.07]	0.33 [0.29; 0.34]	0.96 [0.92; 0.96]
Protein-to-mRNA ratio (PTR)					
SIRT1/mRNA	1.88 [1.81; 1.98]	0.99 [0.90; 1.81]	1.73 [0.95; 2.23]	0.82 [0.80; 0.92]	1.90 [1.88; 2.01]
SIRT3/mRNA	1.87 [1.85; 1.88]	1.10 [0.96; 1.81]	2.09 [1.76; 2.17]	0.92 [0.85; 1.04]	2.45 [2.37; 2.47]
SIRT6/mRNA	1.78 [1.77; 1.80]	2.58 [2.56; 2.69]	2.43 [2.36; 2.64]	2.03 [1.91; 2.03]	2.20 [2.16; 2.23]

Values are presented as median [Q1; Q3]. YA are pooled across sexes; MA and VO are shown separately for males (♂) and females (♀).

**Table 4:** Relative Changes (%) in Sirtuin Markers by Age Transition and Sex.

Marker	Δ MA vs. YA % in ♂	Δ VO vs. MA % in ♂	Δ MA vs. YA % in ♀	ΔVO vs. MA % in ♀
Protein levels (S-)				
sSIRT1	−63.7	−49.4	−10.7	−19.6
sSIRT3	−53.8	−56.7	−31.0	+0.5
sSIRT6	−31.1	−74.5	−32.4	−15.6
mRNA levels				
mRNA-SIRT1	−19.8	−48.5	−24.8	−8.8
mRNA-SIRT3	−23.3	−50.8	−47.8	−1.2
mRNA-SIRT6	−52.2	−66.7	−51.2	−5.0
Protein-to-mRNA ratio (PTR)				
SIRT1/mRNA	−47.3	−8.9	−7.9	+9.8
SIRT3/mRNA	−41.2	−16.4	+11.8	+13.4
SIRT6/mRNA	+44.9	−21.3	+36.0	−9.5

Δ % = ((value_2_ − value_1_)/value_1_) × 100. MA vs. YA compares middle/late-aged (51–84 y) with young adults (≤30 y). VO vs. MA compares very-old (≥90 y) with middle-aged.

**Table 5:** Comparison of Sirtuin Markers in CVD– vs. CVD+ Participants.

Marker	CVD (–) Median [Q1; Q3]	CVD (+) Median [Q1; Q3]	Δ CVD (+) vs. CVD (−) (%)	*p* (M-W)	Effect Size *r*
	Protein levels (S-)				
S-SIRT1	2.01 [1.67; 2.12]	0.44 [0.41; 0.78]	78%	6.3 × 10^−5^	0.84
S-SIRT3	2.12 [2.01; 2.86]	0.62 [0.54; 1.18]	71%	6.3 × 10^−5^	0.84
S-SIRT6	2.55 [2.14; 3.49]	0.70 [0.60; 1.65]	73%	1.7 × 10^−3^	0.69
	mRNA levels				
mRNA–SIRT1	0.94 [0.85; 1.12]	0.55 [0.49; 0.84]	42%	2.9 × 10^−3^	0.63
mRNA–SIRT3	0.88 [0.83; 1.59]	0.64 [0.59; 0.76]	27%	2.6 × 10^−3^	0.63
mRNA–SIRT6	1.01 [0.96; 1.12]	0.34 [0.29; 0.85]	66%	1.0 × 10^−3^	0.69
	Protein-to-mRNA ratio (PTR)				
SIRT1/mRNA	1.90 [1.81; 2.09]	0.86 [0.81; 0.91]	55%	6.3 × 10^−5^	0.84
SIRT3/mRNA	2.32 [1.85; 2.47]	0.95 [0.90; 1.05]	59%	2.2 × 10^−4^	0.77
SIRT6/mRNA	2.23 [2.12; 2.56]	2.03 [1.87; 2.42]	9%	5.6 × 10^−1^	0.11

Δ % = (CVD+ − CVD–)/CVD– × 100; negative values denote decreases in CVD patients. Effect size *r* = |Z|/√(*n*_1_ + *n*_2_); *r* ≥ 0.50 is interpreted as a large effect.

**Table 6:** Preservation of Sirtuin Markers in Very Old vs. Young Adults.

Marker	VO vs. YA, CVD-Free (%)	VO vs. YA, All 23 (%)	*p* (CVD-Free)
Protein levels (S-)			
S-SIRT1	71.7	43.9	0.036
S-SIRT3	69.3	42.8	0.036
S-SIRT6	57.0	36.2	0.036
mRNA levels			
mRNA–SIRT1	68.6	57.9	0.036
mRNA–SIRT3	51.6	45.3	0.036
mRNA–SIRT6	46.4	30.4	0.036
Protein-to-mRNA ratio (PTR)			
SIRT1/mRNA	95.8	67.2	0.053
SIRT3/mRNA	133.6	91.9	0.036
SIRT6/mRNA	124.5	118.2	0.036

Remaining % = (median VO/median YA) × 100.

## Data Availability

All data used in this article are available within the main text. Further inquiries should be directed to the corresponding author.
